# The Development of Categorical Perception of Segments and Suprasegments in Mandarin-Speaking Preschoolers

**DOI:** 10.3389/fpsyg.2021.693366

**Published:** 2021-07-01

**Authors:** Junzhou Ma, Jiaqiang Zhu, Yuxiao Yang, Fei Chen

**Affiliations:** ^1^School of Foreign Languages, Taizhou University, Taizhou, China; ^2^School of Foreign Languages, Hunan University, Changsha, China; ^3^Foreign Studies College, Hunan Normal University, Changsha, China

**Keywords:** development, categorical perception, segments, suprasegments, lexical tones, stops, Mandarin, children

## Abstract

This study investigated the developmental trajectories of categorical perception (CP) of segments (i.e., stops) and suprasegments (i.e., lexical tones) in an attempt to examine the perceptual development of phonological categories and whether CP of suprasegments develops in parallel with that of segments. Forty-seven Mandarin-speaking monolingual preschoolers aged four to six years old, and fourteen adults completed both identification and discrimination tasks of the Tone 1-2 continuum and the /pa/-/p^h^a/ continuum. Results revealed that children could perceive both lexical tones and aspiration of stops in a categorical manner by age four. The boundary position did not depend on age, with children having similar positions to adults regardless of speech continuum types. The boundary width, on the other hand, reached the adult-like level at age six for lexical tones, but not for stops. In addition, the within-category discrimination score did not differ significantly between children and adults for both continua. The between-category discrimination score improved with age and achieved the adult-like level at age five for lexical tones, but still not for stops even at age six. It suggests that the fine-grained perception of phonological categories is a protracted process, and the improvement and varying timeline of the development of segments and suprasegments are discussed in relation to statistical learning of the regularities of speech sounds in ambient language, ongoing maturation of perceptual systems, the memory mechanism underlying perceptual learning, and the intrinsic nature of speech elements.

## Introduction

The development of phonological categories is a complex and long process. Relevant phonological categories in native language are perceived with increasing sensitivity and precision until childhood and even adolescence (Elliott et al., 1981; Hazan and Barrett, 2000; Medina et al., 2010; Ma et al., 2017). The progressive improvement of sensitivity enhances their ability to discern between-category differences (i.e., the differences between two sounds), but it attenuates their sensitivity to within-category differences (i.e., the differences within a sound), preventing irrelevant phonetic variations of the same phoneme accessing their mental lexicon. Consequently, it boosts the efficiency of word recognition and spoken communication. The present study exploited the paradigm of categorical perception (CP), a fine-grained perceptual method, to investigate the perceptual development of phonological categories of both segments and suprasegments in children aged four to six years old.

### Categorical Perception as a Fine-Grained Perceptual Method

Categorical perception, reflecting the fundamental nature of human cognition, has attracted increasing attention in auditory perception of speech sounds (Liberman et al., 1957; Kuhl et al., 2008; Peng et al., 2010), visual perception of colors (Bornstein et al., 1976; Roberson and Davidoff, 2000), and facial expressions (Etcoff and Magee, 1992). In the auditory modality, CP refers to the phenomenon that listeners can perceive continuous acoustic signals as discrete phonological categories and two members within the same category are less discriminable than those from different categories, despite the equivalent acoustic differences between them (Liberman et al., 1957), which offers a sensitive and fine-grained tool to capture the perceptual development of phonological representations. The main signature of typical CP involves the alignment between steep slopes obtained from the identification function and a marked peak obtained from the discrimination function near the categorical boundary (Abramson, 1978). Two tasks are required to assess whether the speech perception is categorical: one is an identification task and the other is a discrimination task. In the identification task, an array of speech stimuli that vary along a continuum are presented. Listeners are required to process acoustic signals and then identify each stimulus, which relies on the effectiveness of auditory and phonological processing. In the discrimination task, listeners are required to judge whether the sound contrasts are identical or different, depending on their linguistic knowledge and psychophysical capacities. Two stimuli from the opposite side of the categorical boundary are defined as between-category pairs, and two from the same side of the boundary are coded as within-category pairs.

Accumulating studies have been undertaken to examine CP, primarily focusing on segments including consonants and vowels (Liberman et al., 1957; Fry et al., 1962; Pisoni and Tash, 1974; Repp et al., 1979; Rosner, 1984), suggesting that the perception of consonants is categorical, characterized by a sharp categorical boundary and a corresponding discrimination peak across or near the boundary. For instance, Liberman et al. (1957) found that listeners perceive synthesized speech sounds which varied along the F2 transition continuum as /b/, /d/, and /g/ only. Their perception changes abruptly from one phonological category to another at certain point along the continuum. In addition, the perception of stops varying along a voice onset time (VOT) continuum is also found to be categorical (Eimas et al., 1971; Medina et al., 2010). For example, Eimas et al. (1971) reported that infants aged one–four months old are sensitive to a 20-ms VOT difference in two speech stimuli around the boundary position on the /b/-/p/ continuum, but they do not show sensitivity to the same difference when the two stimuli are from the same phonological category. However, whether the perception of vowels is categorical or not remains inconclusive (e.g., continuous perception, see Fry et al., 1962; less categorical or quasi-categorical, see Hallé et al., 2004; Altmann et al., 2014).

Recently, there have been increasing interests in the study of CP of suprasegmental features, such as lexical tones in tone languages (Thai — Abramson, 1979; Cantonese — Francis et al., 2003; Mandarin — Xu et al., 2006; Peng et al., 2010; Shen and Froud, 2016). It was found that the perception of level tone contrasts is not categorical while the perception of tone contrasts that involve contour tones remains categorical (Francis et al., 2003; Hallé et al., 2004; Peng et al., 2010). Apart from these behavioral studies, the CP pattern at the suprasegmental level has been confirmed by research adopting electroencephalography. Mandarin-speaking adult listeners provoked larger amplitudes of mismatch negativity to between-category tones than within-category tones (Xi et al., 2010). This supported strong CP of lexical tones among native tone language listeners. Likewise, in the recent studies by Shen and Froud (2016, 2019), both behavioral and electrophysiological correlates of CP of Mandarin lexical tones were specified, demonstrating that between-category tones can be discriminated more easily than within-category tones. Similar findings were also consistently reported by Yu et al. (2014, 2017, 2019).

### Development of Categorical Perception in Speech

Speech perception underlies phonological processing (Ziegler et al., 2009). Our inborn sensitivity to between-category differences plays a vital role in the perception of speech sounds, which requires the capacity to map infinite continuous acoustic signals onto finite phonological categories (Kuhl, 2004). In other words, CP directly associates low-level acoustic cues with high-level phonological categories (Zhao and Kuhl, 2015; Yu et al., 2019), which are exploited to access segment-sized speech units that are further used to identify potential candidates in lexical process. Inaccurate CP of speech sounds of a linguistic environment may hinder the accurate formation of phonological categories, thereby resulting in inferior speech perception (Hakvoort et al., 2016).

Previous research into the development of CP primarily examined one or more phonetic features at either segmental or suprasegmental level in different age groups, suggesting that there exist significant differences between children and adults (Elliott et al., 1981; Burnham, 1986; Flege and Eefting, 1986; Ohde and Sharf, 1988; Nittrouer and Miller, 1997; Walley and Flege, 1999; Medina et al., 2010). However, these studies indeed reveal a prominent developmental pattern, with children progressively approximating the adult-like level in speech perception. For example, Medina et al. (2010) investigated the development of categorical perception of voicing in French-speaking children (nine years old), adolescents (seventeen years old), and adults. Their results revealed significant differences between children and other two groups only in terms of boundary slope, suggesting that it is not until adolescence that they could achieve the adult-like pattern of CP. Moreover, Hazan and Barrett (2000) studied the phonemic categorization of four segment contrasts, including stops and fricatives in children aged six to twelve years old, showing that the boundary slope increases sharply between six and twelve years of age, but they still lack adult-like identification competence. The authors concluded that the development and maturation of CP occur well into the second decade of life. In another study focusing on suprasegmental features in speech processing, Yang and Liu (2012) investigated CP of Tone 1-2 and Tone 1-4 continua in Mandarin-speaking monolingual children aged six to eight years old. Results showed a sharp categorical boundary in the identification task but modest peak in the discrimination task, indicating that children aged eight years old are not able to discriminate both tone continua as well as adults. However, there are some studies showing contradictory results, revealing that there is no significant difference between school-aged children and adults (Sussman and Carney, 1989; Chen et al., 2017). For example, Chen et al. (2017) examined the developmental trajectory of CP of the Tone 1-2 continuum in Mandarin-speaking children aged four to seven years old. Results showed that children aged six years old have reached adult-like identification competence. The inconsistent results are possibly due to the selected speech sound contrasts, experimental tasks, parameters for statistical analysis, or the participants involved in these studies. It, therefore, remains necessary to further examine at what age children can obtain the adult level of CP as they grow up. Furthermore, although numerous prior studies have investigated the development of CP of segments (e.g., vowels and consonants) or suprasegments (e.g., lexical tones), there is a paucity of research into the developmental trajectory of CP of both segments and suprasegments simultaneously in one single study among the same child population, which could deepen our understanding of the perceptual development of phonological categories in children by means of this fine-grained method.

Mandarin is a tone language, which provides us with an ideal opportunity to compare the development of CP of segments and suprasegments within a similar timescale, say, within a syllable, due to the phonemic status of pitch variations over syllables or morphemes in Mandarin (Li et al., 2010). Although acoustic cues, such as duration, amplitude, and vowel quality, contribute to the perception of lexical tones (Whalen and Xu, 1992; Yang, 2015), it is widely recognized that lexical tones in Mandarin are primarily characterized by variations in pitch height or pitch contour within a syllable (Gandour and Harshman, 1978; Gandour, 1981), yielding four different lexical tones: high-level (Tone 1), mid-rising (Tone 2), falling-rising (Tone 3), and high-falling (Tone 4), with the numbers “1,” “2,” “3,” and “4” suggesting different lexical tones (Wang, 1973). For example, /ma1/ (妈) means “mother,” /ma2/ (麻) corresponds to “hemp,” /ma3/ (马) refers to “horse,” and /ma4/ (骂) indicates “to scold” (Chao, 1948). Hence lexical tone constitutes a key factor in lexical retrieval in Mandarin speakers (Cutler and Chen, 1997). In addition, aspiration is a distinctive segmental feature in Mandarin, principally defined by VOT. VOT refers to the temporal interval between burst release and the subsequent vocal cord vibration (Lisker and Abramson, 1964), which is used to differentiate unaspirated stops from aspirated stops in Mandarin as well as voiced stops from voiceless stops in languages like French and Spanish. To be specific, there are three unaspirated stops and three aspirated stops across three places of articulation (i.e., /p, t, k, p^h^, t^h^, k^h^/) in Mandarin Chinese. Although bearing the same tone, alternations in VOT change lexical meanings in usage. For example, /pa1/ (八) means “eight” and /p^h^a1/ (趴) means “lying on one’s stomach.”

To the best of our knowledge, only one study directly compared the differences in the developmental patterns of identification of segmental and suprasegmental features in Mandarin. Xi et al. (2009) investigated the identification of lexical tones and aspiration of stops in typically developing children, suggesting that the developmental pattern of lexical tones differs from that of stops, with children aged six reaching an adult-like level in terms of lexical tone identification while children aged seven still not obtaining adult-like identification abilities for stops. However, it has been confirmed that a hallmark of CP has two basic characteristics: first, identification scores should predict discrimination scores; second, peaks in the discrimination function should align well with the boundary position defined by the identification task (Liberman et al., 1957). In order to attain adult-like phonological abilities, children must not only learn to identify these speech sounds as appropriate phonological categories, but also learn to discriminate speech sound contrasts. In view of this criterion, Xi et al.’s (2009) results seem less convincing due to the lack of discrimination tasks. Therefore, it remains unclear whether the age when children reach an adult-like CP performance varies with speech contrasts at different levels, such as segments and suprasegments.

### The Present Study

The present study sought to explicitly capture the developmental trajectories of segments (i.e., stop consonants) and suprasegments (i.e., lexical tones) by comparing Mandarin-speaking children aged four to six years old with adults across different types of speech sounds. First, based on prior studies (Nittrouer and Miller, 1997; Hazan and Barrett, 2000; Hua and Dodd, 2000; Xi et al., 2009), we hypothesized that children would perceive lexical tones and aspiration of stops categorically and established the phonological categories, whereas they might not obtain the adult-like pattern in terms of some parameters of CP, such as boundary position, boundary width, and discrimination scores in respective between- and within-category sound contrasts. Moreover, we expected the perceptual pattern in children aged six years old would be more similar to that in adults. Finally, we investigated whether the age at which CP in children reached an adult-like level might vary as a function of different speech elements, and we expected that children would show adult-like performance in CP of lexical tones earlier than that of stops.

## Materials and Methods

### Participants

Fifty-one Mandarin-speaking preschoolers were recruited, among whom three failed to complete the experiment due to fatigue, and one did not understand the instruction of the experiment. In the end, forty-seven children participated in the experiment. Although no power analysis was computed, the current sample size was comparable to the studies with similar topics on children’s development of categorical perception (e.g., Hazan and Barrett, 2000; Chen et al., 2017). The young children in this study constituted three age groups according to their age range, namely, the groups of four years old (Male = 11, Female = 3, mean age = 4.7, *SD* = 0.21), five years old (Male = 8, Female = 8, mean age = 5.48, *SD* = 0.35), and six years old (Male = 4, Female = 13, mean age = 6.54, *SD* = 0.32). Notably, our participants received no formal instruction of Pinyin in Mandarin Chinese as well as music training (Zhu et al., 2021). They were all from local kindergartens and shared similar social and economic backgrounds. In addition, fourteen adults as controls (Male = 4, Female = 10, mean age = 21.9, *SD* = 4.5) participated in the experiment. The adults were not musicians and received no music instruction. All children and adults were monolingual speakers of Mandarin Chinese and none had language, speech, or hearing disorders based on their reports from themselves or from their teachers and parents. Each child was given a small gift for their participation. Adults and children’s parents were required to fill in a consent form prior to the experiment, and this experiment was approved by the Ethics Committee of Taizhou University.

### Stimuli

Two types of speech continua were synthesized for the study, including one tonal continuum and one VOT continuum. All speech stimuli were derived in the following manner. An adult native speaker produced Mandarin /i1/ (衣 “clothes”), /pa1/ (八 “eight”), and /p^h^a1/ (趴 “lying on one’s stomach”) 10 times, respectively (44,100-Hz sampling rate, 16-bit digitization). These 30 sounds were assessed by another eight monolingual speakers of Mandarin in order to choose the most natural one for each sound class. These eight judges did not attend the following tests. The selected sounds were then exploited as the natural templates to construct the two types of speech continua, namely, /i1/ (衣 “clothes”) for the tonal continuum, and /pa1/ (八 “eight”) and /p^h^a1/ (趴 “lying on one’s stomach”) for the VOT continuum.

**Figure 1:**
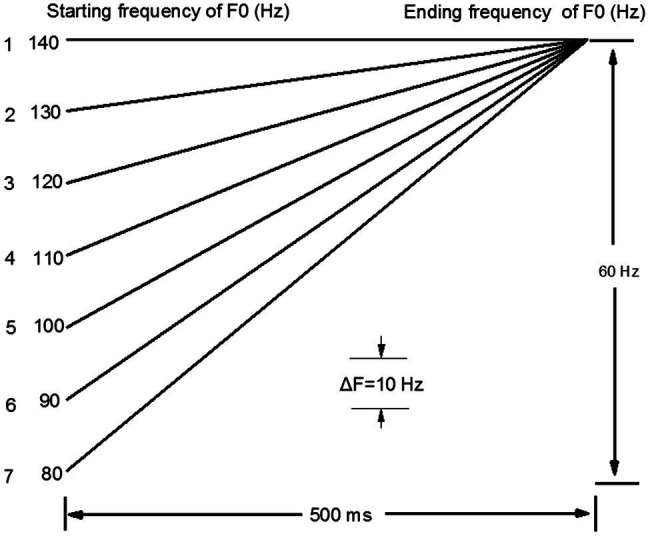
Schematic diagram of the Tone 1-2 continuum.

A tonal continuum ranging from high-level /i1/ (衣 “clothes”) to high-rising (/i2/ 姨 “aunt”) were generated on the basis of the initial template of /i1/ (衣 “clothes”) via applying the pitch-synchronous overlap and added function (Moulines and Laroche, 1995) implemented in Praat (Boersma and Weenink, 2017), with other acoustic cues kept constant. The resynthesizing procedures were that the target syllable was first scaled to 500 ms in duration and 70 dB in intensity, and the pitch contour was fixed to a level tone at 140 Hz; then, a customized script was run in Praat to generate series of the auditory stimuli. The tonal continuum contained seven stimuli with a step size of 10 Hz between adjacent tokens,^1^ which maintained much larger in frequency than the just-noticeable differences in lexical tone perception (Liu, 2013), and what had been used in the previous studies (Xi et al., 2009; Chen et al., 2017). Because the size of pitch variations serves as the greatest determinant of performance (Peretz, 2016), the aim was to maximize the possibility for younger children to capture the physical difference between two speech stimuli. The starting frequency of the stimuli was determined according to the formula 140–10 Hz × (stimulus number – 1). The resultant two endpoints were further judged to be typical exemplars of Mandarin high-level tone (Tone 1) and high-rising tone (Tone 2) by the same eight listeners to guarantee their naturalness and were coded as 1 and 7 in the continuum, respectively. Figure 1 delineates the schematic diagram of the seven stimuli along this tonal continuum.

**Figure 2:**
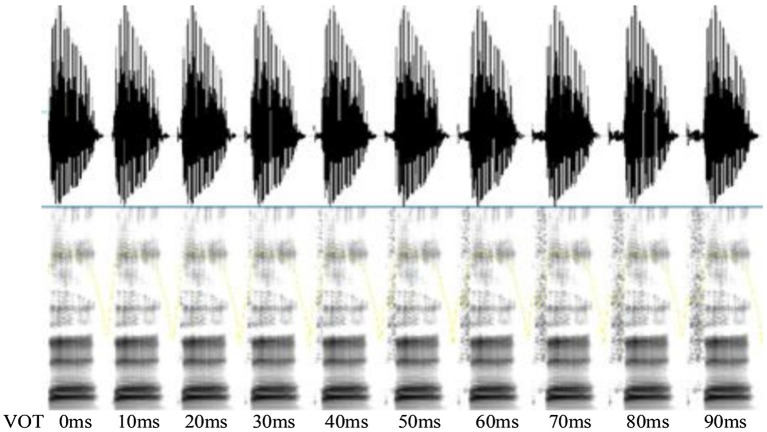
The wideband spectrogram of the /pa1/ - /p^h^a1/ continuum.

To establish the VOT continuum, the progressive cutback and replacement method described in Winn (2020) was adopted. The onset portion of /p/ in the word /pa1/ (八 “eight”) was progressively replaced by the portions of aspiration of /p^h^/ in /p^h^a1/ (趴 “lying on one’s stomach”) in 10-ms increment to create a 10-step VOT continuum ranging from 0 to 90 ms. The range and step size were the same as the criteria used in Xi et al. (2009). The vowel of each stimulus remained constant which was extracted from the word /pa1/ (八 “eight”). The duration of the vowel was 300 ms for all stimuli and all other acoustic cues remained the same. The resultant two endpoints were further judged to be typical exemplars of Mandarin /pa1/ (八 “eight”) and /p^h^a1/ (趴 “lying on one’s stomach”) by the same eight listeners to guarantee their naturalness and were coded as 1 and 10, respectively. Figure 2 depicts the spectrogram of the 10 stimuli along this VOT continuum.

### Procedures

All participants completed two experiments of Experiment A for the tonal continuum and Experiment B for the VOT continuum. Each experiment consisted of two tasks of an identification task and a discrimination task as required in a classic experiment of CP (Xu et al., 2006). The order of the presentation of the two experiments was counterbalanced across participants. Half of the participants finished the tests of the Tone 1-2 continuum on the first day and the VOT continuum on the second day, and the others completed two tasks in a reverse order. There was a time break spanning at least an hour between the identification task and the discrimination task in each experiment, and all participants could take a break at any time during the experiment to avoid the fatigue effect in children.

There was a training session prior to the identification task for both experiments. Two endpoints of each continuum (1 and 7 in the Tone 1-2 continuum and 1 and 10 in the VOT continuum) were played several times to familiarize listeners with the experimental procedures. Following Chen et al. (2017), in Experiment A, upon hearing Stimulus 1, they were instructed to point at the picture on the left (a car driving on a level road) placed on the computer screen, and they were asked to point at the picture on the right (a car driving on a rising road) once Stimulus 7 was played. In Experiment B, they were taught to point at the left picture (Arabic numeral 8 on the paper) if they heard Stimulus 1, and they should point at the right picture (a little boy lying on his stomach) if they heard Stimulus 10. The children could proceed to the next step after they were able to successfully match the stimulus with the corresponding picture. Moreover, before progressing to the formal test blocks, there was a practice block containing Stimuli 1, 2, 4, 6, and 7 in Experiment A and 1, 2, 5, 6, 9, and 10 in Experiment B. Each stimulus in the practice block was played twice randomly, and children were required to identify the Stimuli 1, 2, 6, and 7 in Experiment A and 1, 2, 9, and 10 in Experiment B with an identification rate of no less than 90%. Otherwise they were not allowed to proceed to the formal test blocks. A two-alternative forced-choice paradigm was carried out for the identification task. The 7 stimuli in Experiment A and the 10 stimuli in Experiment B were played eight times randomly, resulting in 56 and 80 trials, respectively. The experimenter clicked the button labeled “第一声” (the orthographic writing of Tone 1 in Mandarin) or “ba” (Pinyin in Mandarin) when the children pointed at the left picture and clicked the button “第二声” (the orthographic writing of Tone 2 in Mandarin) or “pa” (Pinyin in Mandarin) when the children pointed at the right picture. No feedback was provided during the practice block and the formal test blocks. The entire experiment was carried out via ExperimentMFC in Praat.

An AX paradigm was used in the discrimination task (Xu et al., 2006; Peng et al., 2010; Chen et al., 2017). In the training session, children were asked to point at the left picture (a happy face) representing the same pairs when they heard relevant sound pairs (1-1, 7-7 in Experiment A and 1-1, 10-10 in Experiment B); likewise, they were taught to point at the right picture (a sad face) representing different sound contrasts when they heard relevant sound pairs (1-7, 7-1 in Experiment A and 1-10, 10-1 in Experiment B). The purpose of the training session was to ensure that they could successfully match the sound pair with the corresponding picture. The practice block contained 12 pairs in Experiment A (1-1, 2-4, 3-5, and 1-7, repeating three times) and 15 pairs in Experiment B (1-1, 5-7, 6-8, 10-10, and 1-10, repeating three times). In the formal test block, Experiment A contained 17 comparisons, of which 10 pairs consisted of different pairs separated by two steps (different pairs) on the Tone 1-2 continuum in either forward (1-3, 2-4, 3-5, 4-6, and 5-7) or reverse order (3-1, 4-2, 5-3, 6-4, and 7-5), and the other seven pairs consisted of the seven stimuli that paired with itself (identical pairs) on the Tone 1-2 continuum. Analogously, in Experiment B, 26 comparisons were presented randomly. Of these 26 pairs, 16 pairs encompassed different pairs separated by two steps (different pairs) on the VOT continuum in either forward (1-3, 2-4, 3-5, 4-6, 5-7, 6-8, 7-9, and 8-10) or reverse order (3-1, 4-2, 5-3, 6-4, 7-5, 8-6, 9-7, and 10-8), and the remaining 10 pairs consisted of the 10 stimuli that paired with itself (identical pairs) on the VOT continuum. The above comparisons in the discrimination task were repeated five times, yielding 85 pairs and 130 pairs for the tonal and VOT continua, respectively. All stimulus pairs were presented randomly to the participants with an inter-stimulus interval of 500 ms (Peng et al., 2010). Upon hearing each pair, participants were required to decide whether the two sounds in that pair were the same or different by pointing at the picture on the computer screen.

The experimenter would help them click the button labeled “一样” (“same” in Mandarin) when they point at the left picture and click the button labeled “不一样” (“different” in Mandarin) when they point at the right picture. The next stimulus pair was played automatically after the experimenter clicked the button. Adult listeners completed two experiments following the same procedures, except that they did not attend the training session and they clicked the corresponding button by themselves.

### Data Analysis

To examine the development of CP of Mandarin lexical tones and aspiration of stops, the position of the categorical boundary, the width of the categorical boundary, and the discrimination score were obtained following the procedure in the previous studies (Peng et al., 2010; Zhang et al., 2017).

The identification score of each stimulus was calculated as the percentage of responses with which participants judged the stimulus as Tone 1 or Tone 2 in Experiment A and /pa/ or /p^h^a/ in Experiment B. The boundary position and boundary width were assessed by Probit analysis of the identification curve of each participant (Finney, 1971). The boundary position was defined as the 50% crossover point of the two identification curves, and the boundary width was defined as the linear distance between the 25th and 75th percentiles.

With respect to the discrimination task, all the trials were divided into five comparison units in Experiment A and eight comparison units in Experiment B. Each comparison unit comprised four types of pairwise comparisons (AB, BA, AA, and BB), where AB and BA represented different pairs and AA and BB depicted identical pairs. Adjacent comparison units included overlapping AA or BB pairs. The discrimination score (*P*) of each sound pair was computed by using the equation described in Xu et al. (2006).

$$P=P\left(“S”\mathrm{|S}\right)\times P\left(S\right)+P\left(“D”\mathrm{|D}\right)\times P\left(D\right)$$

In the above equation, *P*(“S”|S) and *P*(“D”|D) refer to the percentage of the “same” responses (“S”) to all the “same” pairs (S), and of the “different” responses (“D”) to all the “different” pairs (D), while *P*(S) and *P*(D) represent the probabilities of the “same” and “different” trials in each comparison unit, respectively, which are 50% in this study. Further, the between-category discrimination score (*P*_bc_) and within-category discrimination score (*P*_wc_) of each participant were calculated for the Tone 1-2 continuum and the VOT continuum. *P*_bc_ refers to the average score of stimulus pairs straddling the categorical boundary, and *P*_wc_ denotes the average score of stimulus pairs within the same category on the basis of identification boundary position obtained in the identification task.

## Results

### CP of the Tone 1-2 Continuum

#### Identification and Discrimination Curves

**Figure 3:**
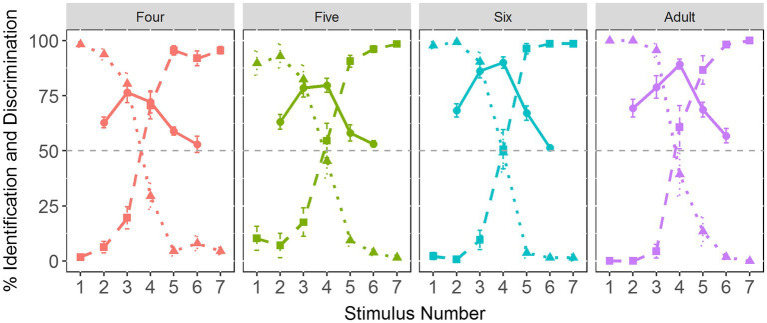
The identification and discrimination curves across four age groups. The dotted line represents Tone 1 responses. The dashed line indicates Tone 2 responses. The solid line refers to discrimination curves.

Figure 3 demonstrates the identification and discrimination curves for the Tone 1-2 continuum. It can be seen that the categorical boundary aligns well with the discrimination peak for each age group, suggesting that children show similar CP patterns of the Tone 1-2 continuum to adults.

#### Position and Width of Categorical Boundary

**Figure 4:**
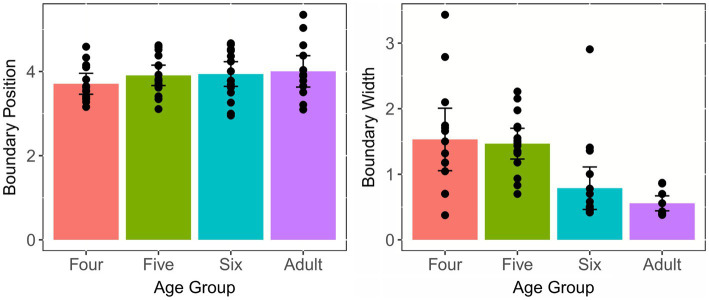
Means and standard errors of the boundary position and width for the Tone 1-2 continuum across age groups. Error bars: ±1 standard error.

The means and standard errors (SEs) of the boundary position and width across age groups are shown in Figure 4, which clearly illustrates that the boundary position is similar across age groups, whereas children aged four and five have much wider boundary width as opposed to adults and children aged six. A one-way ANOVA, with group as the independent factor and boundary position as the dependent factor, was performed to examine the effect of age on the position of categorical boundary. The results revealed that there was no statistically significant difference between groups [*F*(3, 57) = 0.81, *p* = 0.494, *η*^2^ = 0.041], suggesting that three child groups had comparable positions of categorical boundary with adults. In addition, a similar analysis was conducted to determine the effect of age on the width of categorical boundary. The results demonstrated that the main effect of group reached a significant level [*F*(3, 57) = 10.73, *p* < 0.001, *η*^2^ = 0.361]. *Post-hoc* analysis using the Tukey’s method showed that adults and the six-year-olds had significantly narrower boundary widths than the four- and five-year-olds (*p*s < 0.01), whereas the boundary width between the adults and the six-year-olds were not significantly different (*p* = 0.68); the same pattern was observed between the four-year-olds and the five-year-olds (*p* = 0.99). This indicated that children aged six years old achieved adult-like degree of CP of the Tone 1-2 continuum.

**Figure 5:**
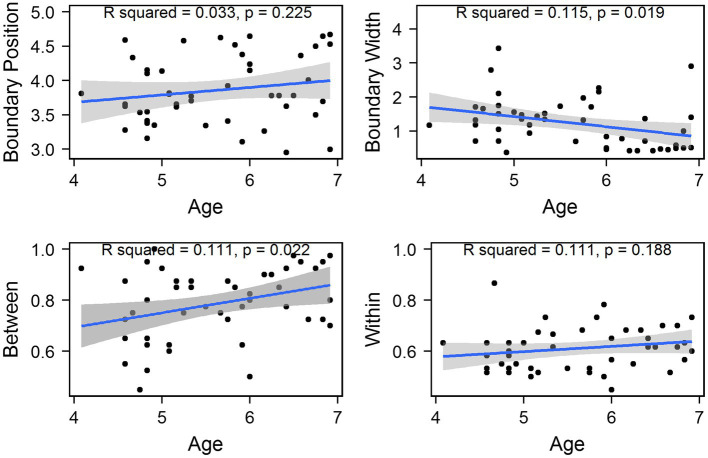
Values of all tokens for each child plotted against children’s chronological age for the Tone 1-2 continuum. The shadow area represents the 95% confidence interval of the best-fit line from linear regression models.

Two simple linear regressions were performed to examine whether children’s chronological age could significantly predict the boundary position and width, respectively. As shown in Figure 5, the results revealed that the former model was not significant [*F*(1, 45) = 1.513, *p* = 0.225]. However, the latter model was significant [*F*(1, 45) = 5.894, *p* = 0.019] and explained 11.5% of the variance in the boundary width. It was found that age significantly predicted the narrowing boundary width (*β*_1_ = −0.298, *p* = 0.019). This suggested that children continuously fine-tuned their boundary width to approximate adult-like patterns, thereby more likely to map relevant phonetic variations onto phonological representations stored in long-term memory.

#### Discrimination Score

**Figure 6:**
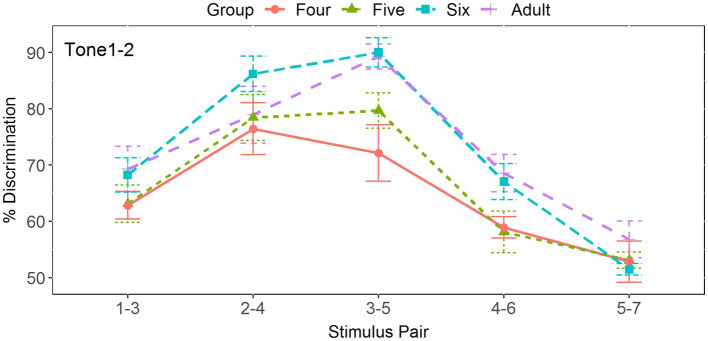
Two-step discrimination scores for the five pairs in the tonal continuum across age groups.

Figure 6 shows that the score for the four-year-old children reached its maximum at Pair 2-4, straddling its corresponding boundary position at 3.71. The mean score at this position is 72% for the four-year-olds, 80% for the five-year-olds, 90% for the six-year-olds, and 89% for the adults. In order to examine whether there was significant difference between these four groups, a one-way ANOVA was performed. The results revealed no significant differences at this position [*F*(3, 57) = 1.099, *p* = 0.357, *η*^2^ = 0.055], whereas the discrimination peaks for the adults and the five- and six-year-old children were located at Pair 3-5, straddling the boundary position from 3.91 to 4.0. The result of one-way ANOVA showed a significant effect of age group [*F*(3, 57) = 6.323, *p* < 0.001, *η*^2^ = 0.25]. Pairwise comparisons using the Tukey’s method demonstrated that the score for the four-year-old children was significantly lower than that for the adults and six-year-old children (*p*s < 0.01), but no other significant differences were observed between other groups (*p*s > 0.05), suggesting that children aged five years old achieved adult-like discrimination abilities in terms of discrimination peak.

**Figure 7:**
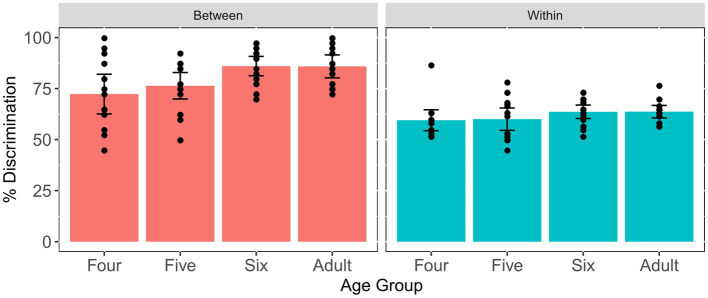
Means and standard errors of the between- and within-category discrimination scores for the Tone 1-2 continuum across age groups. Error bars: ±1 standard error.

In order to explore whether there existed age differences in the between-category discrimination score and the within-category discrimination score, we further divided the five comparison units into between-category comparison units and within-category comparison units for each individual. Figure 7 displays the means and SEs of the between-category discrimination score and within-category discrimination score for each age group. It can be seen that the four- and five-year-old groups have relatively lower between-category discrimination scores, while all four groups have similar within-category discrimination scores. The results of one-way ANOVA revealed significant age differences in terms of the between-category discrimination score [*F*(3, 57) = 4.76, *p* = 0.005, *η*^2^ = 0.2]. Subsequent pairwise comparison showed that there was significant difference between the four-year-old children and adults (*p* = 0.024) and the six-year-old children (*p* = 0.015). In addition, with respect to the within-category discrimination score, one-way ANOVA reported no significant age differences [*F*(3, 57) = 1.209, *p* = 0.315, *η*^2^ = 0.06]. These results indicated that all three child groups performed analogously to adults in the discrimination of within-category stimuli and achieved an adult-like level at age five for the discrimination of between-category stimuli.

Furthermore, two simple linear regressions were performed to investigate the relationship between the between- and within-category discrimination scores and children’s chronological ages, respectively. The first model significantly accounted for 11% of the variance [*F*(1, 45) = 5.615, *p* = 0.022] and children’s chronological age significantly predicated the improvement of between-category discrimination scores (*β*_1_ = 0.056, *p* = 0.022); however, the model for the within-category discrimination and age was not significant [*F*(1, 45) = 1.789, *p* = 0.188], as shown in Figure 5, indicating that children’s sensitivity to between-category differences significantly improved with age, yet their sensitivity to within-category differences remain constant in preschoolers.

### CP of the VOT Continuum

#### Identification and Discrimination Curves

**Figure 8:**
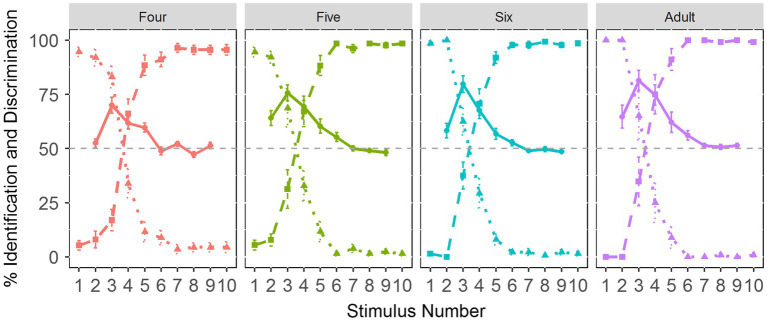
The identification and discrimination curves of the VOT continuum across age groups. The dotted line represents /pa1/ responses. The dashed line indicates /p^h^a1/ responses. The solid line refers to discrimination curves.

Figure 8 illustrates the identification and discrimination curves of the VOT continuum. As can be seen, there remains a clear alignment between the categorical boundary and the discrimination peak, indicative of CP of the VOT continuum in each age group.

#### Position and Width of Categorical Boundary

**Figure 9:**
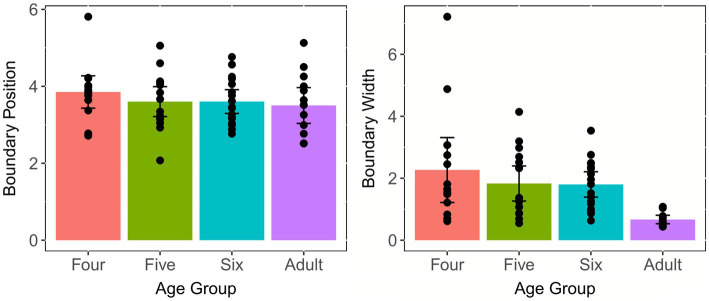
Means and standard errors of the boundary position and width for the VOT continuum across age groups. Error bars: ±1 standard error.

Figure 9 shows the means and SEs of the position of categorical boundary and the width of categorical boundary for the VOT continuum. It is observed that the four groups have similar boundary positions. However, adults have much narrower boundary widths than the three child groups.

Two similar one-way ANOVAs were conducted to examine the effects of age on boundary position and boundary width. The results showed that there were no significant age effects for the position of categorical boundary [*F*(3, 57) = 0.618, *p* = 0.606, *η*^2^ = 0.032]. However, a significant age effect was found for the width of categorical boundary [*F*(3, 57) = 5.297, *p* = 0.003, *η*^2^ = 0.218]. Tukey’s *post-hoc* analysis showed that adults had significantly narrower boundary width than three child groups (*p*s < 0.05), indicating that children had not reached an adult-like level in the perception of aspiration of stops by the age of six years old.

**Figure 10:**
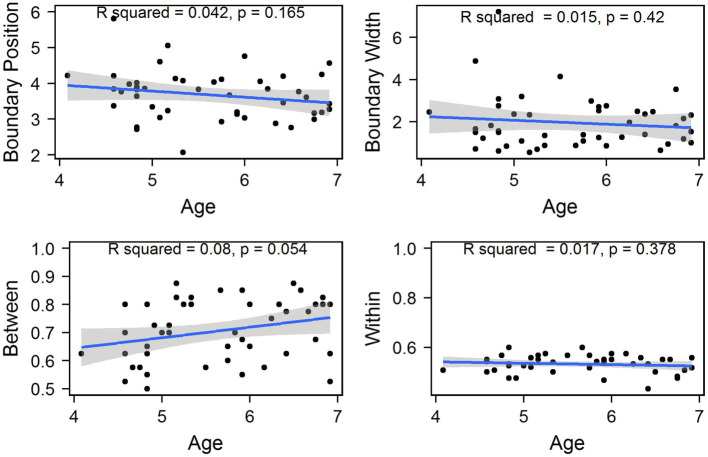
Values of all tokens for each child plotted against children’s chronological age for the VOT continuum. The shadow area represents the 95% confidence interval of the best-fit line from linear regression models.

Additionally, two simple linear regressions were carried out to examine if age significantly predict the boundary position and boundary width. The regression analysis showed that both models were not significant [*F*(1, 45) = 1.989, *p* = 0.165; *F*(1, 45) = 0.662, *p* = 0.42], suggesting that both boundary position and width did not change as a function of age in preschoolers, as shown in Figure 10.

#### Discrimination Score

**Figure 11:**
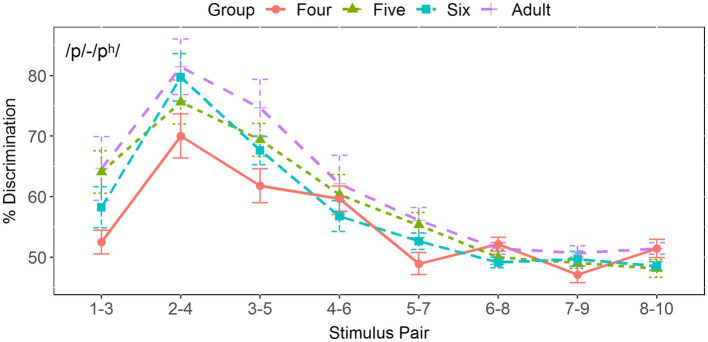
Two-step discrimination scores for the eight pairs in the VOT continuum across age groups.

Figure 11 depicts that the score for all four groups peaks at Pair 2-4, straddling from 3.5 to 3.85. The mean discrimination peak is 70% for the four-year-olds, 76% for the five-year-olds, 80% for the six-year-olds, and 81% for the adults. The results of one-way ANOVA revealed no significant age effect at its maximum at this position [*F*(3, 57) = 1.562, *p* = 0.209, *η*^2^ = 0.076].

**Figure 12:**
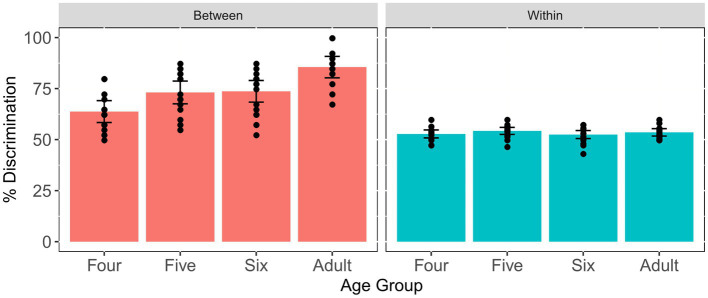
Means and standard errors of the between- and within-category discrimination scores for the VOT continuum across age groups. Error bars: ±1 standard error.

The eight comparison units were further divided into between-category pairs and within-category pairs to calculate the between- and within-category discrimination scores, respectively. Figure 12 demonstrates that the between-category discrimination score for the adults is higher than that for the three child groups, whereas all four groups have comparable within-category discrimination scores. The results of one-way ANOVA showed that no significant age differences were found among four groups in terms of the within-category discrimination score [*F*(3, 57) = 0.903, *p* = 0.445, *η*^2^ = 0.045]. However, there was significant age difference in the between-category discrimination score [*F*(3, 57) = 11.5, *p* < 0.001, *η*^2^ = 0.377]. Tukey’s *post-hoc* comparisons revealed that there were significant differences between adults and three child groups (*p*s < 0.01), with adults having significantly higher scores than all child groups. This indicated that the perception of within-category stimuli was similar between children and adults, yet children still had not reached an adult-like level in the discrimination of between-category stimuli along the VOT continuum by age six.

With respect to the relationship between discrimination scores and chronological ages, two linear regression models were built. The results revealed that both models were not significant either [*F*(1, 45) = 3.93, *p* = 0.054; *F*(1, 45) = 0.791, *p* = 0.378], and children’s chronological ages could not significantly predict the between- and within-category discrimination scores, suggesting that both between- and within-category discrimination scores did not change during this period, as shown in Figure 10.

## Discussion

The aim of the present study was to examine the CP of lexical tones and stops in Mandarin-speaking children in an attempt to investigate the fine-grained perceptual development of phonological categories. The results revealed that children could perceive both lexical tones and aspiration of stops categorically by the age of four years old. In addition, the boundary position and within-category discrimination scores did not depend on age, regardless of types of speech elements. The boundary width reached the adult-like level at age six for lexical tones, but not for stops. The between-category discrimination score improved with age and achieved the adult-like level at age five for lexical tones, but still not for stops even at age six. These results were discussed in detail as below.

### Protracted Development of Phonological Categories

The results suggest that the basic CP pattern was acquired quite early in young children (e.g., Medina et al., 2010; Chen et al., 2017). Although our finding echoed earlier studies of CP in both normal and dyslexic children (Xi et al., 2009; Zhang et al., 2012; Chen et al., 2017), it ran contrary to the previous studies of English-speaking children who showed no CP of Mandarin Tone 1-2 continuum at age six to eight years old (Yang and Liu, 2012), indicating that continuous exposure to tone language plays a vital role in the development of CP of lexical tones and aspiration of stops. Children develop their ability to form new phonological categories based on the distribution of tokens in ambient languages (Best et al., 2016), that is, they could extract the regularities of linguistic input via statistical learning. However, the exact time when children could perceive lexical tones and aspiration of stops following a categorical fashion remains unknown due to the absence of much younger infants, as suggested by many previous studies that infants’ ability to perceive speech sounds into different phonological categories is an indispensable part of the biological component of human beings and such ability is operative at an unexpectedly early age (Eimas et al., 1971). Nevertheless, our findings indicate that they have established the phonemic inventory of tones and stops by age four. In other words, children have similar phonemic inventory of Tone 1, Tone 2, /pa/, and /p^h^a/. This, in turn, could help account for why previous studies based on adults’ perceptual coding showed earlier acquisition of tones and stops than studies using instrumental analysis (e.g., Hua and Dodd, 2000; Ma et al., 2018; Xu Rattanasone et al., 2018; Yang, 2018; Ma and Zhou, 2019).

The development of phonological categories is a protracted process. Although children as early as four years old could perceive Mandarin lexical tones and aspiration of stops categorically, it still takes much time to become fully mature, the length of which varies depending on various factors as reported in the previous studies (Best et al., 2016; Chen et al., 2017; Basu et al., 2018). This corroborates findings from the previous studies (Hazan and Barrett, 2000; Xi et al., 2009; Chen et al., 2017) by uncovering that children aged six years old, without explicit Pinyin instruction, have adult-like identification competence for the Tone 1-2 continuum, but children aged six have not reached the same level as adults for the VOT continuum. Younger children do not have the same ability to process speech sounds or prevent those irrelevant phonetic variations entering the mental lexicon, nor do they have the same ability to activate the phonological representations stored in long-term memory. Consider that we ensured that all children could identify the endpoints with an identification rate of no less than 90%, it is unlikely that such age effects were a result of attentional deficits in children. Children’s sharpening of categorical boundaries between ages of five and six might be explained by different mechanisms. One plausible account is that language-specific phonological categories are learned through prototypes. Members of a phonological category are attracted by the most frequent exemplar (the perceptual magnet) of the category (Kuhl et al., 2008). Another account attributes the emergence of language-specific phonological representations to the development of language-specific categorical boundaries, which are obtained by modifying universal boundaries to phonological categories in a specific language environment. However, the finding of children’s sharpening boundary slopes (i.e., narrowing boundary width) does not corroborate with the Native Language Magnet theory (Kuhl et al., 2008), because their improvement is mainly due to the increase in the identification score of stimuli near the boundary, not of those exemplars at the end of the continuum. Increasing exposure to specific ambient language environment sharpens the sensitivity around the categorical boundaries between phonological categories and attenuates the sensitivity to the prototypes, consistent with the previous studies of the perceptual reorganization that occurs between 6 and 12 months of age, when infants reshape the universal boundaries to language-specific boundaries (Aslin and Pisoni, 1980). Children’s continuous attunement to language-specific phonological categories is based on consistent exposure to the distribution of linguistic input in the ambient language.

In addition, the difference between younger children and adults might be related to children’s ongoing maturation of perceptual systems (Hazan and Barrett, 2000). The development of auditory sensitivity is long-lasting and continues to improve even in adolescence (Howell and Williams, 2004; Huyck and Wright, 2007). Huyck and Wright (2007) investigated the development of temporal-interval discrimination in children, adolescents, and adults, revealing significant differences between children and adolescents, but comparable performance between adolescents and adults. This might explain the late development of CP, indicative of the complex and long-lasting nature of the development of speech perception.

### Earlier Development of Tones Than Stops

Crucially, children’s improvement of identification performance at age six and discrimination performance at age five for lexical tones provides supporting evidence for the dual-process model (Fujisaki and Kawashima, 1971). From the perspective of this model, short-term memory can be divided into a continuous auditory short-term store and a categorical phonetic short-term store, respectively. The former is subject to fast decay and is of critical importance in discriminating within-category stimuli, while the latter remains more stable as a result of its “contact” with mental representations in long-term memory, which is tightly linked to the discrimination of between-category stimuli (Xu et al., 2006). The overall discrimination ability is determined by the combination of auditory memory and phonetic memory in the decision-making process after decay. Both types of memory co-exist in the discrimination task, while participants operate merely in the phonetic short-term memory in the identification task (Fujisaki and Kawashima, 1971). Our tasks required participants to decide whether the contrasts paired by two stimuli were identical or different for discrimination. If the two stimuli were from different categories (i.e., between-category stimuli), listeners tended to rely on their phonetic memory code in the decision-making process; instead, if the two stimuli were from the same category (i.e., within-category stimuli), the auditory short-term memory would be employed to make a decision. Representations in short-term categorical memory could be permanently stored in long-term memory due to continuous perceptual learning (Xu et al., 2006). Considerable exposure to the tone language environment leads to the storage of tonal category information in long-term memory, which facilitates the categorical phonetic short-term memory due to their “contact.” It is therefore reasonable to infer that the identification and discrimination abilities of lexical tones experience a progressive developmental trajectory as a result of continuous exposure to ambient language. However, the development of stops does not parallel with lexical tones as manifested by the absence of improvement of identification and discrimination abilities of stops for children by age six. Tones are more widely distributed in the input as compared to stops (Wang, 1973). The “contact” for lexical tones would be far more frequent than that for stop consonants for Mandarin-speaking children. Thus, tones are more likely to be stored readily in the phonological representations than stops in long-term memory. Therefore, children are able to acquire adult-like level of identification and discrimination abilities for lexical tones earlier than stops.

Alternatively, the cue-duration hypothesis could provide plausible account for the earlier maturation of identification and discrimination abilities for lexical tones as compared to stop consonants. Fujisaki and Kawashima (1970) explored the perception of consonants and vowels and they found that the critical acoustic cue that distinguish vowels (i.e., formant frequency) is comparatively longer, extending the entire stimulus; however, those cues employed to distinguish stops (i.e., VOT and formant transition) are shorter, which cannot be well stored in memory. In the current study, as measured through Praat, the duration of lexical tones also extends the whole syllable /i/ to 500 ms, while the VOT of stops ranges from 0 to 90 ms in the syllable initial position. Therefore, children are more likely to attend to critical information in longer sounds, resulting in earlier development of identification and discrimination abilities of lexical tones.

Apart from the above-mentioned memory-based interpretations, the varying timeline of the identification and discrimination abilities of lexical tones and aspiration of stops could also be possibly attributable to the intrinsic nature of the two types of speech sounds. Lexical tones are primarily characterized by slowly changing F0, whereas aspiration of stops is mainly manifested by fast shifts of the temporal cue of VOT. This being the case, it could be challenging for preschoolers to process fast changing information as opposed to slowly changing information due to the immaturation of their perceptual capacities (Xi et al., 2009). Additionally, the differences in phonological saliency of lexical tones and stops could also play a role in the speech development. Hua and Dodd (2000) investigated the acquisition order of speech elements in Mandarin Chinese systemically, suggesting that Mandarin lexical tones are acquired earlier than vowels, followed by syllable-final consonants and finally syllable-initial consonants. They proposed the phonological saliency hypothesis to account for the acquisition order of speech elements, the saliency of which depends on the number of options they have in specific phonological systems. It is believed that higher phonological saliency contributes to earlier acquisition of speech sounds. In Mandarin, there are four lexical tones and twenty-one syllable-initial consonants. Each syllable must carry a tone which is used to distinguish lexical meanings, while the presence of a syllable-initial consonant is optional (Wang, 1973). Consequently, the fewer options of lexical tones and the more options of syllable-initial consonants lead to higher saliency for lexical tones and lower saliency for consonants, as evidenced in our current results of CP among children. Children are more likely to use the powerful mechanism of statistical learning to extract the regularities of tones as opposed to stop consonants in the speech input.

It is worthwhile to note that other factors may also contribute to the varying developmental trajectories of CP of lexical tones and aspiration of stops. For example, the specific distributional features of phonetic information in their caregivers could affect the developmental path. In addition, the pictures used in the experiment could be potentially another confounding factor. The pictures that depicted tonal contours did not tap into children’s lexical representations, whereas the two pictures for the VOT continuum might be opposite as they hinted meanings to listeners. It is therefore reasonable to postulate that through different pictures for stimulus presentation, the perception of stops would be easier than tones, because the former involves explicit top-down knowledge. However, it is intriguing that children still perceived Tone 1-2 continuum categorically and performed much better than stop consonants. It is possible that children are quite familiar with /yi1/ and /yi2/ in daily spoken communication. Among them, the most frequently used are /yi1/ “one”, /yi1/ “clothes,” /yi1/ “doctor,” and /yi2/ “aunt.” As a result, relevant acoustic signals could still tap into children’s lexical representations without the help of pictures expressing semantics, because children may allocate extensive attention to the categorical prototypes associated with each tone. Further, as implied by Zhou et al. (2012), the preliterate children are skillful in mapping between prosody and semantics to resolve speech act ambiguities. It is of interest to further examine whether and how the selection of pictures may affect the outcome of categorical perception, especially for younger children.

Our findings also lead to the question of whether and how the immature CP will affect children’s tone and stop production. It is likely that younger children are perceptually less sensitive to the subtle acoustic changes. Consequently, their immature perception results in non-adult-like production. Addressing this question could deepen our understanding of the relationship between perception and production. Future studies are recommended to recruit children of a wider age range to capture the fine-grained nature of CP across multiple types of speech contrasts such that a clearer scenario of the development of CP of Mandarin Chinese can be obtained. Furthermore, neural studies are anticipated in light of the varying developmental patterns in the CP of segments and suprasegments in children.

## Conclusion

The current study revealed that the perceptual development of phonological categories is a protracted process, and CP of lexical tones and aspiration of stops did not parallel with each other, probably fully adult-like at age six for tones and well beyond six for stops, although children could perceive both types of speech continua in a categorical manner by age four. Our findings provide further evidence for the improvement and varying developmental trajectories of segments and suprasegments as a result of statistical learning of regularities in ambient language, maturation of perceptual use of acoustic information, the memory mechanism underlying perceptual learning, and the intrinsic nature of different speech elements.

## Data Availability Statement

The raw data supporting the conclusions of this article will be made available by the authors, without undue reservation.

## Ethics Statement

The studies involving human participants were reviewed and approved by the Ethics Committee of Taizhou University. Written informed consent to participate in this study was provided by the participants’ legal guardian/next of kin.

## Author Contributions

JM formulated the research questions, collected data, conducted statistical analysis, and wrote the draft of the paper. JM, JZ, and FC designed the experiment and synthesized speech materials. JZ, YY, and FC contributed to the draft of the paper. FC supervised the whole process. All authors have approved the final version of the manuscript.

### Conflict of Interest

The authors declare that the research was conducted in the absence of any commercial or financial relationships that could be construed as a potential conflict of interest.

## Footnotes

^1^We performed a pilot study in which an 11-step tonal continuum and a 10-step VOT continuum were used. Unfortunately, seven of the 10 children aged four to five years old were reluctant to collaborate with the experimenter and failed to finish the two experiments mainly due to serious fatigue, frustration, or unwillingness, although they were required to finish Experiment A on the first day and Experiment B on the following day (or vice versa), and they were also free to take a break anytime in each experiment. It was therefore of necessity to strike a balance between the number of speech stimuli (or the number of experimental trials) and the burden that younger children had to face in the experiment such that they could finish the two experiments collaboratively and successfully. We finally chose the tonal continuum with seven steps.
